# The Cure of Diabetes

**Published:** 1883-01

**Authors:** 


### THE CURE OF SACCHARINE DIABETES.

In a paper by Dr. G. Félizet, read before the Academy of Sciences, August 14, says the *Journal d'Hygiene*, the author claims to have discovered a remedy for a disease usually regarded as incurable—saccharine diabetes. The author states that he has succeeded in putting an end to glycosuria artificially produced in animals, and that the medicine that suppresses that artificial glycosuria will likewise cure diabetes in a few weeks or months. There exists, says he, a bond of union between artificial glycosuria, intermittent diabetes and confirmed diabetes, and that bond is irritation of the rachidian bulb. It is not, then, in masking the disease by submission to the severities of a regime exempt from bread, feculents, sugar, etc., that we succeed in curing it, but by tapping the very source of the production of sugar, that is to say, by suppressing the irritation of the bulb. Bromide of potassium, by the elective action of sedation that it exerts on the functions of the bulb, suppresses the effects of such irritation with a rapidity that is often surprising, and, in large and repeated doses, cures the diabetes.

